# The Zebrafish Anillin-eGFP Reporter Marks Late Dividing Retinal Precursors and Stem Cells Entering Neuronal Lineages

**DOI:** 10.1371/journal.pone.0170356

**Published:** 2017-01-20

**Authors:** Meret Cepero Malo, Anne-Laure Duchemin, Luca Guglielmi, Eva Patzel, Saadettin Sel, Gerd U. Auffarth, Matthias Carl, Lucia Poggi

**Affiliations:** 1 Centre for Organismal Studies, Heidelberg University, Heidelberg, Germany; 2 Department of Cell and Molecular Biology, Medical Faculty Mannheim, Heidelberg University, Mannheim, Germany; 3 The David J Apple Center for Vision Research, Department of Ophthalmology, Heidelberg University Hospital, Heidelberg, Germany; University of Michigan, UNITED STATES

## Abstract

Monitoring cycling behaviours of stem and somatic cells in the living animal is a powerful tool to better understand tissue development and homeostasis. The tg(*anillin*:*anillin-eGFP*) transgenic line carries the full-length zebrafish F-actin binding protein Anillin fused to eGFP from a bacterial artificial chromosome (BAC) containing Anillin cis-regulatory sequences. Here we report the suitability of the Anillin-eGFP reporter as a direct indicator of cycling cells in the late embryonic and post-embryonic retina. We show that combining the *anillin*:*anillin-eGFP* with other transgenes such as *ptf1a*:*dsRed* and *atoh7*:*gap-RFP* allows obtaining spatial and temporal resolution of the mitotic potentials of specific retinal cell populations. This is exemplified by the analysis of the origin of the previously reported apically and non-apically dividing late committed precursors of the photoreceptor and horizontal cell layers.

## Introduction

Reliable detection and direct monitoring of cell division events in the living organism is crucial if we want to understand proliferative behaviours in embryonic and post-embryonic tissues. To this aim, the generation of zebrafish transgenic lines as reporters for cell cycle activity has proven to be an invaluable resource for today’s biomedical research [1,2]. The F-actin binding protein Anillin is an important actomyosin regulator and midbody component, and crucial player in the cell cycle of proliferating cells [3–5]. Subcellular localization of the Anillin protein is cell cycle dependent—being restricted to the nucleus during late G1-, S- and G2-phase, released to the cytoplasm during nuclear envelope breakdown at prophase, and enriched in the contractile ring and midbody during late M-phase [6,7]. Such dynamic characteristics make Anillin-eGFP reporters particularly suitable for direct visualisation and quantification of variations of cell division behaviours such as mitosis progression and daughter cell midbody inheritance [7–10]. We have recently reported the expression of *anillin* in the early proliferative neuroepithelium of the developing zebrafish retina [10]. Establishing a BAC-based *anillin*:*anillin-eGFP* transgenic line where expression of Anillin is fused to the enhanced green fluorescent protein (Anillin-eGFP) allowed us to recapitulate temporal and spatial dynamics of both *anillin* expression and Anillin protein subcellular localisations *in vivo* [10]. Using this tool, we uncovered that asymmetric midbody inheritance is predictive of daughter cell developmental fate [10]. Here we assess the suitability of the transgene as readout for retinal cell mitotic potentials in late embryonic and post-embryonic stages of retinal maturation. The extent to which late “committed” retinal precursors and even early post-mitotic retinal cells are capable of re-entering the cell cycle remains poorly understood. For instance, Müller Glia have been shown to be able to re-enter mitosis, both in normal conditions and in response to injury, with important implications for retinal regenerative potentials [11–14]. Additionally, there have been reports of cells of the differentiated horizontal and photoreceptor cell layer, which already express post-mitotic markers of differentiation, yet are able to re-enter the mitotic cycle in the late maturing retina [15–17]. On the one hand, it was postulated that these cells correspond to late fate-committed precursors, endowed with the capability to undergo terminal symmetric divisions to generate more of the same kind of retinal types [15–17]. In contrast, studies in the young mouse retina have reported that fully differentiated horizontal cells can give rise to metastatic retinoblastoma [18], therefore attracting substantial attention towards the potential plasticity of this retinal cell type [19,20]. Here, we assess expression of the Anillin-eGFP reporter as a versatile indicator of proliferative activities in distinct populations of fate-restricted precursors of the late maturing central retina and stem cell niche of both late embryonic and larval stage. Our analysis underscores the advantages of the Anillin-eGFP reporter and provides insights into the possible developmental origin of apical and non-apical committed precursors of the late maturing central retina.

## Results and Discussion

### Anillin-eGFP expression marks dividing cells, IKNM and midbody positioning in the maturing retina

**Fig 1:**
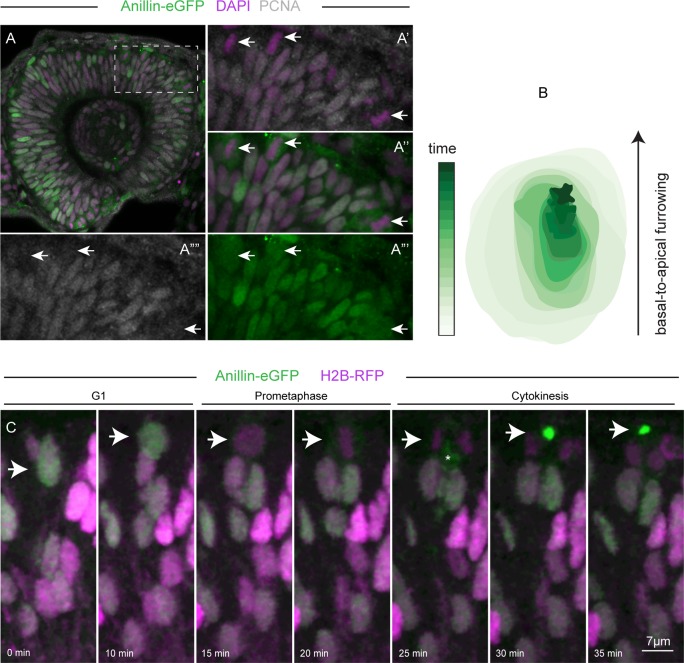
Anillin-eGFP dynamics in cycling cells. (A) At 28 hpf the retinal neuroepithelium is highly proliferative, as detected by the Proliferating Cell Nuclear Antigen (PCNA, grey), a marker of proliferative activity, highly expressed in the G1, G2 and S phases of the cell cycle [23]. (A’-A””) magnifications of the area delineated by the dotted square in (A). Both Anillin-eGFP (green) and PCNA (grey) are expressed in the nuclei (magenta) of retinal progenitor cells during the G1, S and G2 phase of the cell cycle. Notably, different intensities of Anillin-eGFP are consistent with the dynamic expression of Anillin, which is lower during G1 and higher during G2 [7]. The white arrows in (A’-A””) point at PCNA negative cells in metaphase, showing characteristic localisation of Anillin-eGFP expression in the cytoplasm upon nuclear envelope breakdown. (B) A contour plot of Anillin-eGFP at the contractile ring depicts basal-to-apical cleavage furrow progression in time. (C) Frames extracted from S1 Movie (min: minutes), describing Anillin-eGFP (green, white arrow) release in the cytoplasm at prometaphase, its accumulation at the basal side of the cell body (asterisk) at the beginning of cytokinesis, and around the apically positioned midbody at the end of cytokinesis. Cell nuclei (magenta) were labelled with mRNAs encoding H2B-RFP as previously described [10].

Early in development the whole retinal neuroepithelium is proliferative (Fig 1A–1A””). Nuclear expression of the Anillin-eGFP reporter depicts interkinetic nuclear migration (IKNM) of dividing retinal progenitor cells as they undergo G1, S and G2 phases of the cell cycle, spanning the entire apical basal axis of the epithelium [10,21] (S1 Movie and Fig 1C). Mitoses occur at the apical most side and begin with Anillin-eGFP release in the cytoplasm upon nuclear envelope breakdown in early pro-metaphase (S1 Movie and Fig 1C). Accumulation of Anillin-eGFP at the basal side of the nucleus demarcates the beginning of cytokinesis [8], which proceeds with the basal-to-apical cleavage furrow ingression and positioning of the midbody remnant at the cell apical domain [10,22] (S1 Movie and Fig 1B and 1C).

**Fig 2:**
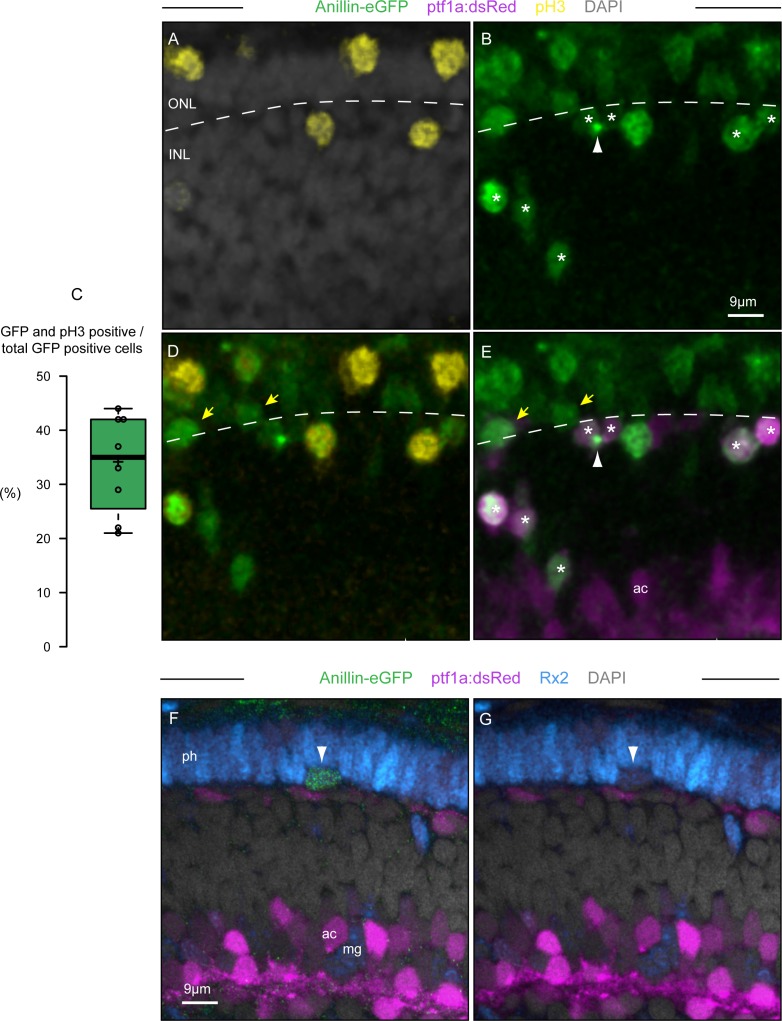
Anillin-eGFP marks all cycling cells of the maturing retinal layers. (A-E) Optical section from a z-stack (frontal view) through the retina of a 48 hpf, *anillin*:*anillin-eGFP/ptf1a*:*dsRed* transgenic zebrafish that have been counterstained with DAPI (A) and pH3-antibody (A,D). (C) Only 34% of the Anillin-eGFP positive cell nuclei are pH3 immunoreactive at 48 hpf (n = 8 retinas). The black horizontal line shows the median while the crosses show the average. The box limits indicate the 25th and 75th percentiles as determined by R software; whiskers extend 1.5 times the interquartile range from the 25th and 75th percentiles, data points are plotted as open circles. Asterisks in (B) and (E) highlight cells that are (*ptf1a*)dsRed (magenta) and Anillin-eGFP (green) positive, only few of which are also pH3 positive (yellow in D). The arrow in (B) and (E) points at the midbody between dividing daughter cells (asterisk) during late cytokinesis. (F,G) Optical section from a z-stack (frontal view) through the retina of an 60 hpf, *anillin*:*anillin-eGFP/ptf1a*:*dsRed* transgenic zebrafish that have been counterstained with DAPI (grey) and Rx2-antibody (blue). The arrowhead points at the cycling, Anillin-eGFP positive and Rx2-immunoreactive cell located at the base of the outer limiting membrane. ONL, outer nuclear layer; INL, inner nuclear layer; ac, amacrine cell; ph, photoreceptors; mg, Müller glia cell bodies (intermingling with the ac bodies).

As maturation of the central retina advances the three retinal cell layers become distinguishable, with the nuclei of cone and rod photoreceptor precursors becoming allocated to the outer-most layer (ONL), amacrines and horizontal interneurons to the inner nuclear layer (INL), and retinal ganglion cells (RGCs) as well as some displaced amacrines to the inner-most ganglion cell layer (GCL). At this time of development, only few cells express *anillin* as most retinal progenitors exited the cell cycle or are in their terminal division [10]. Time-lapse imaging in the retina of double *anillin*:*anillin-eGFP/atoh7*:*gap-RFP* transgenic zebrafish, where expression of membrane-bound (gap43) RFP marks all three maturing cell layers [24], shows that IKNM becomes progressively restricted to the INL, perhaps as a consequence of less cell “crowding” in this retinal domain [24] (S2 Movie). Consequently, mitotic activities can also occur at non-apical locations within the retinal epithelium [17] (S2 Movie, arrow). It was suggested that such non-apical divisions are characteristics of committed precursors of horizontal interneurons, which are able to re-enter mitosis whilst expressing markers of post-mitotic neuronal differentiation [15–17]. To specifically label mitotic divisions we used anti-phosphorylated histone H3 (pH3), which mark cells in the late G2/M phase of the cell cycle [25]. As expected from previous work [16,17], immunohistochemistry with anti-pH3 antibodies detects both apically and non-apically dividing cells across the maturing central retinal epithelium at 48 hours post fertilization (hpf) (Fig 2A and 2D). Notably, Anillin-eGFP reporter expression marks cells in the G2-, M-, S- and late G1-phase of the cell cycle as well as in the final stages of cytokinesis (Fig 1 and S1 Movie) [7,10]. As a consequence, only 34% of the total population of cells with cell cycle activity (i.e. that are Anillin-eGFP positive) could be detected by the late G2/M-phase marker pH3 at 48 hpf (Fig 2C). Thus, expression of the Anillin-eGFP reporter allows both monitoring and examining dynamic behaviours of such reported mitotically active committed precursors when they are highlighted by expression of retinal cell-specific transgenes. We combined *anillin*:*anillin-eGFP* with the *ptf1a*:*dsRed* transgene, which is normally expressed in post-mitotic horizontal and amacrine cell precursors [26]. In line with previous work [15–17], some (*ptf1a*)dsRed-positive cells located in the presumptive horizontal cell layer were both eGFP-positive and pH3-immunoereactive, indicating that they undergo mitosis at non-apical locations (Fig 2D and 2E). In addition, dsRed-positive cells with cycling activity could also be revealed by Anillin-eGFP reporter expression at more basal locations within the neuroepithelium (asterisks in Fig 2B and 2E). This observation is suggestive of previously reported IKNM of horizontal cell precursors [27] before they undergo another mitotic division [28]. Finally, the Anillin-eGFP reporter also makes it possible to label and visualize cytokinesis events such as cleavage furrow and midbody positioning between daughter cells (arrowhead in Fig 2B and 2E). This may provide further relevant information as to the differences in modes of division of apically and non-apically dividing cell types.

Many Anillin-eGFP positive cells were also detected in the ONL, which were dsRed-negative (Fig 2B, 2D and 2E). This observation is consistent with previous work reporting mitotic activity of committed photoreceptor cell precursors [17,29]. Some of these eGFP-positive cells were located more sub-apically, in proximity of the horizontal cell layer (yellow arrows in Fig 2D and 2E), suggesting that they might correspond to distinct retinal cell populations. It has been reported that Müller glia cells can function as late-stage retinal progenitors of the photoreceptor lineage, with their cell bodies occasionally migrating to the horizontal cell layer to divide asymmetrically, generating one photoreceptor and one Müller glial cell [12]. To assess the existence of such Müller glial cell divisions, we carried out immunolabelling with Rx2, which allows labelling cell bodies of photoreceptor as well as Müller glial precursors of the INL (Fig 2F and 2G [30]). Some Rx2 immunoreactive cells could be indeed observed with their cell bodies located sub-apically, between the outer limiting membrane and the horizontal cell layer. Intriguingly, these Rx2 immunoreactive cells also express Anillin-eGFP (arrowhead in Fig 2F and 2G), supporting the idea that Müller glial precursors contribute to the population of non-apically dividing cells at 60 hpf. Thus, Anillin-eGFP provides a useful tool for detecting “retinal layer-allocated divisions” reported in the late maturing central retina. In addition, the Anillin-eGFP reporter complements and extends the use of other cell cycle markers such that distinct retinal cell division behaviours like the extent of IKNM within the neuroepithelium, the progression through cytokinesis, or daughter cell midbody positioning can be examined [7,10,22].

### The Anillin-eGFP reporter allows studying late neuron generation in the post-embryonic retina

**Fig 3:**
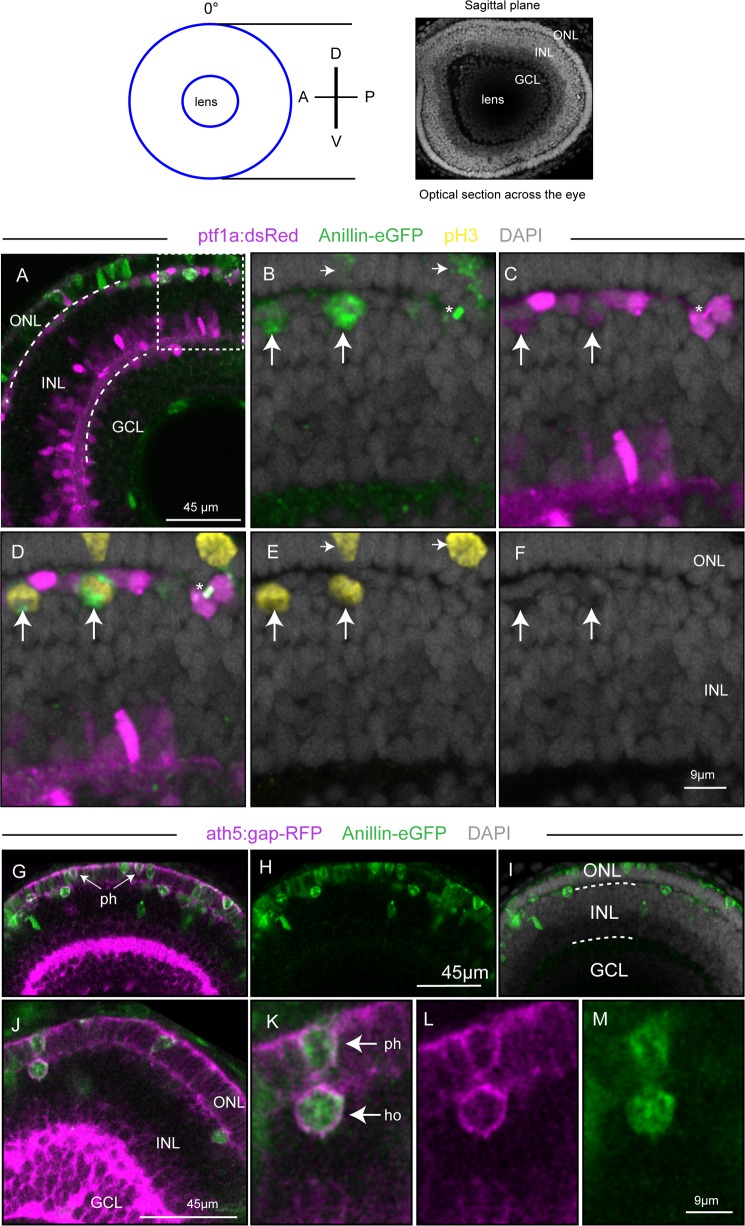
Visualisation of Anillin-eGFP labelled cycling cells in the post-embryonic central retina at 3 dpf. Top panel: view of the central retina in the sagittal plane (0°, eye facing the viewer). The blue circles represent an optical z-section across the eye corresponding to the blue line in the top panel of Fig 4. (A-F) Optical section from a z-stack through the retina of an 3 dpf, *anillin*:*anillin-eGFP/ptf1a*:*dsRed* transgenic zebrafish taken in the sagittal plane. (B-F) Magnification of the area delineated by the dotted rectangle in (A). The vertical arrows in (B-F) point at dividing horizontal cells that are (*ptf1a*)dsRed positive (magenta in C,D), Anillin-eGFP positive (green in B) and pH3 positive (yellow in D,E), and at their corresponding nuclei (F). The asterisk in (B-D) highlights an Anillin-eGFP labelled midbody between daughter cells in late cytokinesis, not depicted by pH3. The horizontal arrows in (B) and (E) point at two Anillin-eGFP positive (green in B) and pH3 positive (yellow in E) mitotic nuclei in the ONL. (G-I) and (J-M) represent two different optical sections from a z-stack through the retina of an 3 dpf, *anillin*:*anillin-eGFP/atoh7*:*gap-RFP* transgenic zebrafish in the sagittal plane. The arrows in (G) and (K) point at *atoh7*:*gap-RFP* expressing (magenta) photoreceptor and horizontal cell precursors that are also Anillin-eGFP (green) positive, indicating that they are in the cell cycle. GCL, ganglion cell layer; INL, inner nuclear layer; ONL, outer nuclear layer; ph, photoreceptor cell; ho, horizontal cell.

We next assessed whether the Anillin-eGFP reporter can also be used to mark layer-restricted cell division activities in the post-embryonic retina. It is thought that by 3 days post fertilization (dpf) the differentiation of the central retina is complete [31], and that lifelong growth exhibited by teleost fish is sustained by the addition of newborn retinal cells originating from a stem cell niche within the peripheral ciliary marginal zone (CMZ) [32–34]. Yet, both apically and non-apically dividing committed precursors have been reported, which are suggested to play a prominent role in the late maturing central retina [17]. To detect such late dividing precursors, we first analysed z-stacks of optical sections taken in the sagittal plane (lateral view, eye facing the viewer as shown in the top panel of Fig 3), through the retina of an 3 dpf, *anillin*:*anillin-eGFP/ptf1a*:*dsRed* transgenic zebrafish (Fig 3A and 3F). While both transgenes co-localise with pH3 in the horizontal cell layer (vertical arrows in Fig 3B and 3F), only Anillin-eGFP reporter expression detects dividing horizontal cells in the late cytokinesis phase (asterisk in Fig 3B–3D). In addition, mitotic activities could be observed in the ONL where differentiated photoreceptors are located (horizontal arrows in Fig 3B and 3E). We then analysed the double transgenic *anillin*:*anillin-eGFP/atoh7*:*gap-RFP* line, which expresses the membrane-bound (gap43)-RFP [24] also in photoreceptor cell precursors [17,35] (S2 Movie). Cells co-expressing Anillin-eGFP and (gap43)-RFP could be detected in the photoreceptor cell layer (Fig 3G and 3M), as expected from previous work reporting proliferative activity of committed photoreceptor precursors at 3 dpf [17]. Thus, these data support the idea that retinal cells of the central retina can still undergo mitosis at 3 dpf, at least in the horizontal and photoreceptor layers.

### Layer-restricted generation of late retinal neurons might be characteristic for the CMZ

**Fig 4:**
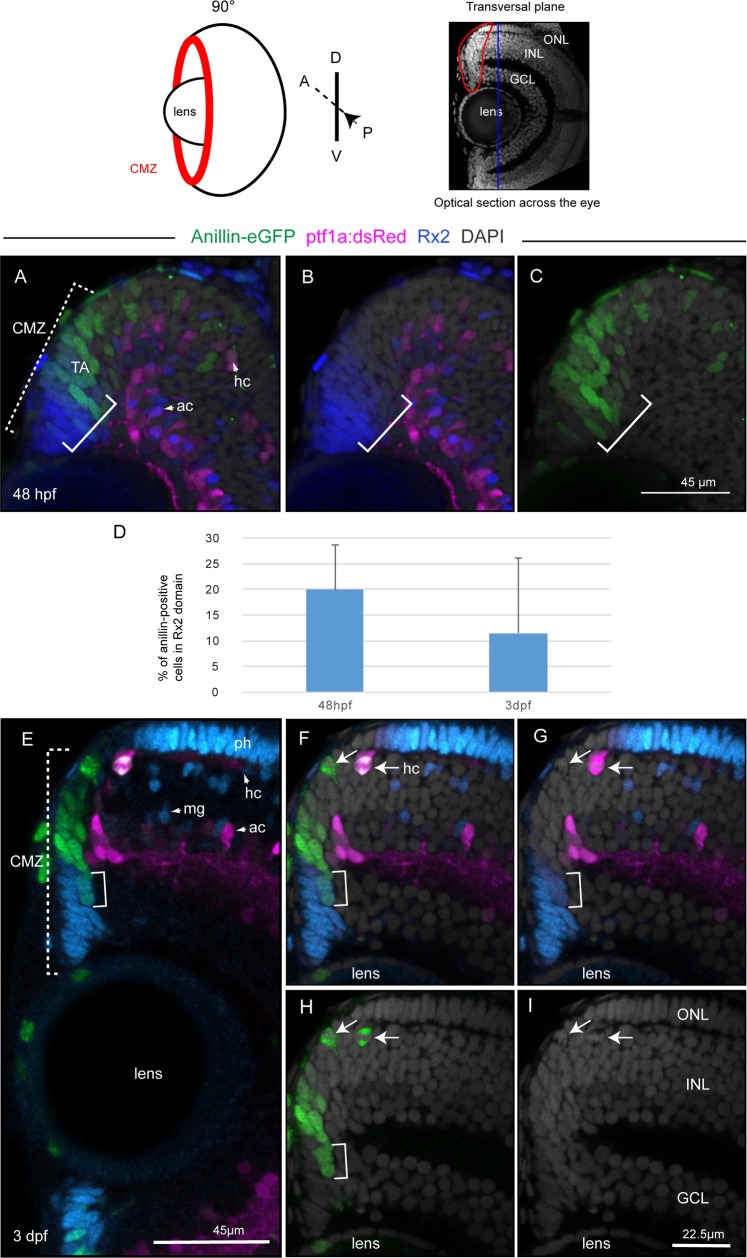
Anillin-eGFP in the CMZ marks transit-amplifying progenitors entering restricted neuronal lineages. Top panel: view of the retina in the transversal plane (eye position 90° relative to the viewer). The blue line across the retina corresponds to the sagittal view (optical z-section) in the top panel of Fig 3. (A-C) and (E-I) represent two optical sections from confocal z-stacks through the retina of a 48 hpf (A-C) and 3 dpf (E-I) zebrafish taken in the transversal plane. The squared dotted bracket in (A) and (E) delineates the CMZ domains. The squared bracket in (A-C) and (E-H) delineates cells within the CMZ that are both Anillin-eGFP (green) and Rx2 immunoreactive (blue). (D) As the stem cell niche develops, the percentage of Rx2 immunoreative cells that are also Anillin-eGFP positive in the CMZ domain decreases over time from 20% to 11,4%. The arrows in (F-I) point at two dividing, Anillin-eGFP (green) positive cells and at their nuclei (I). (F,G) One of the two cells is also (*ptf1a*)dsRed positive and non-apically located. Ph, photoreceptors; ac, amacrine cells; hc, horizontal cells; mg, Müller glial cell. ONL, outer nuclear layer; INL, inner nuclear layer; ONL, outer nuclear layer.

While these studies demonstrate the occurrence of late mitoses in the central retina, they leave the unresolved question about how these dividing cells relate to newly generated retinal cells within the CMZ, which are thought to be predominant by 3 dpf [31–33]. We therefore used expression of the Anillin-eGFP reporter to resolve cell division activities in the CMZ. Slow-cycling stem cells, located in the extreme periphery of the CMZ, are characterized by expression of the retina-specific homeobox 2 (Rx2) transcription factor, a marker for multipotent neural stem cells (NSCs) [30]. NSCs give rise to faster cycling, transient amplifying (TA) progenitor cells in the so-called transient amplifying zone [30], which is located in a more central domain of the CMZ. These highly proliferative TA progenitors might be restricted in their developmental fate potentials [34] and are required for the constant production of the pools of lineage-committed precursor cells, which are located in the central-most portion of the CMZ [32,36]. To resolve these distinct cellular domains, unlike the sagittal plane (lateral view) commonly used in previous works, we took z-stacks of optical confocal sections in the transversal plane (embryo mounted frontally, as shown in the top panel of Fig 4 as compared to the top panel in Fig 3) across the central retina. This view conveniently allows simultaneously resolving Anillin-eGFP positive and Rx2 immunoreactive cells of the CMZ domains (dotted squared bracket in Fig 4A and 4E) and the differentiated neuronal layers of the central retina, defined by expression of the *ptf1a*:*dsRed* transgene in the INL (Fig 4A and 4E). Here, distinct expression of the Anillin-eGFP reporter in all proliferative active cells allows discerning domains of retinal NSCs (green and Rx2 positive) and TA progenitor cells (green and Rx2 negative) within the CMZ of both embryonic- (Fig 4A and 4C) and larval-stage (Fig 4E and 4I) zebrafish. This enables, for example, quantifying dynamics of both NSCs and TA progenitor cells development and distributions in space and developmental time (Fig 4C, 4D and 4H).

**Fig 5:**
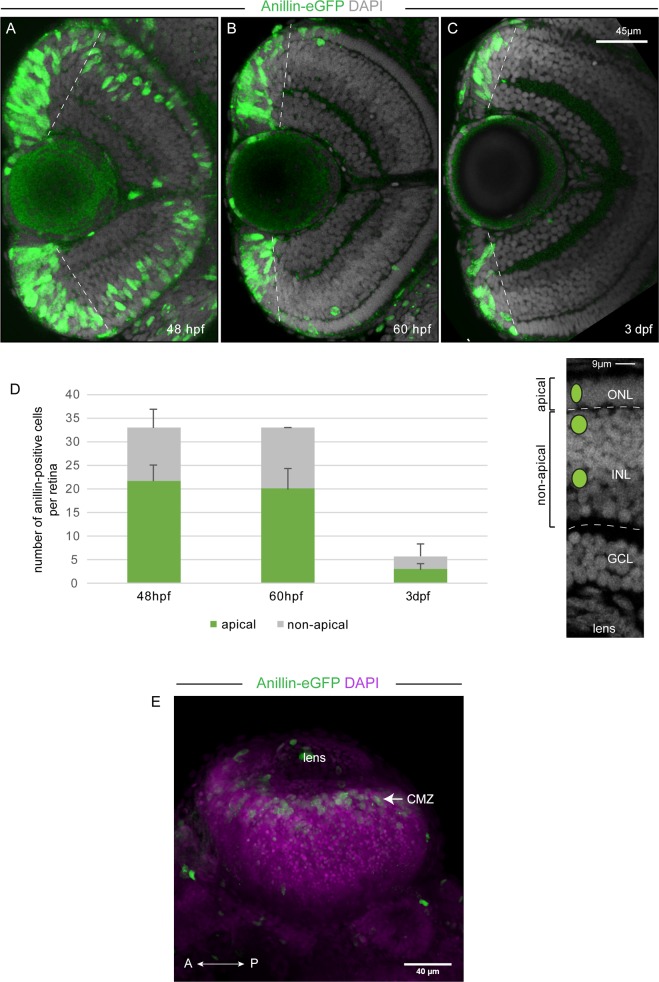
Anillin-eGFP labelled cell division activity is restricted to the CMZ at 3 dpf and at 5 dpf. (A-C) Optical section from a z-stack taken in the transversal plane (frontal view as represented in the top panel of Fig 4) through the central retina of an *anillin*:*anillin-eGFP* transgenic zebrafish fixed at 48 hpf (A), 60 hpf (B) or 3 dpf (C). Counterstaining with DAPI (grey) marks the cell nuclei and the three retinal cell layers. (D) Left: Anillin-eGFP positive cells were counted, which were allocated to the three retinal nuclear layers of the central retina (excluding the CMZ delineated by the dotted line). Anillin-eGFP positive cells were counted per embryo (48hpf, n = 3 apical = 22±4, non-apical = 11±4; 60 hpf, n = 3, apical = 20±4, non-apical = 13±0; 3dpf, n = 3, apical = 3±3, non-apical = 3±1,5). Right: Overlay of a confocal image with a schematic view representing the different locations of Anillin-eGFP positive cells within the central retina: apical, in the outer nuclear layer (ONL) and non-apical in the inner nuclear layer (INL). Error bars represent standard deviation. GCL: ganglion cell layer. (E) Two-photon z-stack projection (z-sections 1 μm apart) of a retina from a 5 dpf zebrafish showing Anillin-eGFP positive cells (green) in the CMZ. The cell nuclei (magenta) were labelled with DAPI. The image represents a dorsal view.

To resolve divisions of TA progenitor cells entering committed precursor cell lineages, we analysed expression of the Anillin-eGFP and (*ptf1a*)dsRed reporters from the *anillin*:*anillin-eGFP;ptf1a*:*dsRed* transgenes when visualised in the transversal plane (as in the top panel of Fig 4). Firstly, expression of the Anillin-eGFP reporter reveals the occurrence of divisions at apical and non-apical locations within the central-most part of the CMZ where the newborn post-mitotic cells are located [33,36] (arrows in Fig 4F–4I). Furthermore, co-expression of the (*ptf1a*)dsRed reporter indicates that non-apically positioned divisions occur in horizontal cell precursors that are likely generated within the CMZ and are already positioned in the horizontal cell layer (Fig 4F and 4I). This observation raises the possibility that layer-restricted mitoses at 3 dpf, as detected from sagittal views, are hallmarks of neurogenic activity of the CMZ. Accordingly, most mitoses observed at 3 dpf should be localized predominantly within or around the CMZ. To assess this, we analysed the Anillin-eGFP reporter expression in the retina from z-stacks taken in the transversal plane at 48, 60 hpf and 3 dpf (Fig 5A–5C). Apically and non-apically located, Anillin-eGFP positive cells could be detected in the central retina of all three analysed stages (Fig 5A–5D). However, we could barely record any Anillin-eGFP positive cell outside the CMZ of 3 dpf retinas (Fig 5C and 5D).

Thus, examining expression of cell-type specific transgenes in combination with Anillin-eGFP reporter expression in the transversal plane is an excellently suited prerequisite for accurate resolving of distinct spatially and temporally distributed modes of cell division of committed precursors in the CMZ and embryonic central retina. This analysis might allow assessing, for example, the possibility that particular modes of layer-restricted divisions provide a generally utilized mechanism whereby rapid and efficient integration of newborn cells into pre-existing retinal circuits is achieved both in the late maturing central retina and post-embryonic CMZ. Alternatively, layer-restricted divisions might be a characteristic of distinct cell populations, perhaps arising within the developing CMZ domain. Combining the Anillin-eGFP reporter with available stem cell markers and cell lineage tracing methods [34] will enable acquiring further relevant information related to spatial-temporal dynamics of the CMZ. It is noteworthy that Anillin-eGFP expression can still be detected at larval stages later than 4 dpf (Fig 5E). Therefore, this reporter may well be an important tool for also understanding events that characterise CMZ behaviour during homeostasis.

In conclusion, the Anillin-eGFP reporter serves as valuable tool whereby spatial-temporal dynamics of endogenous Anillin expression can be examined in physiological condition, even at larval stages of development. This allows concomitantly resolving spatial-temporal patterns of cell division of distinct retinal cell populations in the maturing central retina and CMZ. The Anillin-eGFP reporter complements and extends the use of other established zebrafish reporters for cycling cells [1,2], providing information on the dynamics of cell division behaviour such as, for example, cytokinesis progression and midbody positioning, both *in vivo* and in the fixed animal. The reporter’s applications are certainly not restricted to the analysis of cell dynamics during maturation of the central retina and post-embryonic CMZ, but can be extended to any tissue of interest. In addition, the reporter will be an important tool to study Anillin putative roles in many relevant cellular processes such as asymmetric cell division, cell polarity, metastatic transformation and migrating ability [5, 37–43]

## Materials and Methods

### Animals and ethic statement

Zebrafish (*Danio rerio*) breeding/raising was done as previously described [44–46]. The adult fish were maintained at 28°C and kept in the fish facility built according to the German Animal Welfare act (Tierschutzgesetz §11, Abs. 1, Nr. 1, Haltungserlaubnis) and in accordance with European Union animal welfare guidelines. Lines used in this study were generated in the zebrafish wildtype background (AB/AB or AB/WIK). The fish facility is under the supervision of the local representative of the Animal Welfare Agency.

### Fish strains

The *anillin*:*anillin-eGFP/atoh7*:*gap-RFP* and *anillin*:*anillin-eGFP*/*ptf1a*:*dsRed* double transgenic lines were generated by crossing *anillin*:*anillin-eGFP* transgenic fish [10] with *atoh7*:*gap-RFP* [24] and *ptf1a*:*dsRed* [26] transgenic, respectively. Embryos carrying both transgenes were screened for expression of the RFP and GFP reporters using an Olympus MVX10 macrofluorescence binocular.

### Immunostaining

For all immunostainings, zebrafish embryos were fixed in 4% PFA, washed in 1xPBT 3 times for 5 minutes and digested in a Trypsin- 2.5% EDTA solution for 30 minutes (on ice). Subsequently, they were washed in PBT 4 times for 10 minutes. The first solution change was performed on ice. To avoid unspecific binding of the antibodies, the embryos were blocked in a solution containing 10% goat serum, 1%BSA and 0.8% Triton in PBT for minimum 1 hour at room temperature. Blocking solution was replaced by the primary antibody mix chicken anti-GFP antibody [Life technologies, diluted 1:200)] and either rabbit anti-rx2 [Charles River (diluted 1:200)], rabbit anti-PH3 [Millipore, (diluted 1:400)] or mouse anti-PCNA antibody [Millipore (diluted 1:250)] and DAPI (diluted 1:1000) in the following solution: 1% goat serum, 1% BSA and 0.8% Triton. Embryos were incubated at 4°C for 2 overnights with primary antibodies. After washing in PBT for several hours, incubation with the secondary antibodies [anti- chicken antibody, Alexa 488 [Dianova (diluted 1:250)] and anti-rabbit antibody, Alexa 647 [Thermo Fischer (diluted1:250)] and DAPI, diluted 1:1000 in 1% goat serum, 1% BSA and 0.8% Triton solution was carried out for 2 overnights.

### Imaging

Confocal time-lapse imaging was performed as previously described [10,26,45–47]. Embryos were mounted onto imaging plates [35 mm Glass-bottom Microwell dish (P35G-1.5-10-C, MatTek)] in 1% low-melting agarose. Embryos were oriented with the help of bend femtoloader tips (Eppendorf). Orientations were lateral (eye facing the viewer) for sagittal view and frontal (eye positioned 90 degree with respect to the viewer) for transversal view (top panels of Figs 3 and 4). 1 to 10 embryos were mounted per plate. Confocal images were taken using a Leica 63X 1.15 NA oil-immersion objectives and Leica Application Suite (LAS) software. 30–60 μm Z-stacks were taken with 1 μm thick optical sections. For the panels in Fig 1, and the S1 Movie, mosaic distribution of H2B-RPF/Anillin-eGFP positive cells was obtained with the transplantation method as previously described ([10] and Dudczig et al., JOVE, in press), allowing for cellular resolution of Anillin-eGFP imaging. Time-lapse imaging of Anillin-eGFP dynamics was performed with maximum 5-minute intervals for up to 20 hours with optical sections of 1 μm. Imaging of Anillin-eGFP, DAPI and PCNA shown in Fig 1A was performed with a sequential scan using both two photon (for the Anillin and the DAPI) and confocal (for the PCNA) microscopy. For the Fig 5E, two-photon imaging across the retina of a 5 dpf zebrafish was performed to image Anillin-eGFP and DAPI.

### Image processing and statistical analysis

Analysis of furrowing was made on Fiji drawing the contour of Anillin-eGFP reporter expression at each time-point and extracting the contours. Quantification of Anillin-eGFP and pH3 positive cells as well as Anillin-eGFP and Rx2 positive cells was done in Volocity 6.0.1 (PerkinElmer). Counting of the DAPI-labelled cell nuclei was done on 8–12 Z-sections across a confocal stack taken in the transversal plane (frontal view) (as indicated in Fig 4). For the quantification of both pH3 and Anillin-eGFP positive cell nuclei in the central retina, cell counting was performed only carried out when an ONL, INL and GCL could be unambiguously identified (thereby mostly excluding cells of the CMZ delineated by the dotted line in Fig 5). For the quantification of Anillin-eGFP and Rx2 positive cells of Fig 4, counting was performed within the CMZ-only. Brightness and contrast were adjusted for all channels evenly using Fiji or Adobe Photoshop CS4. All ratios and standard deviations were calculated with Excel (MS Excel 2013). The boxplots were made using the webpage http://boxplot.tyerslab.com/ (based on R-software).

## Supporting Information

S1 Movie *In vivo* dynamics of Anillin-eGFP cellular distribution during cell division and IKNM. Time-lapse confocal imaging through the retina of a 28 hpf. All time points are z-projections of confocal stacks imaged in lateral view (retina facing the eye). The white arrow points at a cell nucleus in G1, which undergoes mitosis at the apical surface. The yellow arrow points at a cell nucleus in G2, which migrates towards the basal surface. Mosaic distribution of Anillin-eGFP positive cells has been obtained transplanting cells from an *anillin*:*anillin-eGFP* transgenic embryo injected with mRNAs encoding H2B-RFP (to label all cell nuclei) into a non transgenic host as previously described ([10,26,45–47] and Dudczig et al., JOVE, in press).
(AVI)

S2 Movie Anillin-eGFP reporter expression highlights progressive confinement of IKNM within the inner nuclear layer. Time-lapse confocal imaging through the retina of a double transgenic *anillin*:*anillin-eGFP/atoh7*:*gap-RFP* zebrafish embryo (30 to 50 hpf) showing temporal and spatial dynamics of IKNM and cell mitoses. The white arrowhead points at a cell undergoing non-apical mitosis as the maturing ganglion cell layer (GCL), inner nuclear layer (INL) and outer nuclear layer (ONL) become distinguishable. All time points are z-projections of confocal stacks imaged in lateral view (retina facing the eye).
(MOV)
